# Hospital and Institutional News

**Published:** 1913-12-06

**Authors:** 


### HOSPITAL AND INSTITUTIONAL NEWS.

### OUR CHRISTMAS PRIZES.

The Hospital this year, as usual, will publish a Special Appeal Number, exhibiting what the spirit of personal service achieves for those who give and those who receive. In order to bring home to the public what is the spirit of personal service within the institutions themselves, we offer five prizes of two guineas for the best illustrated account or one guinea for the best account without illustrations of "How to Spend Christmas in a Hospital," from the five points of view of the Resident Medical Staff, the Secretariat, Matron, Sister, or Nurse, competitors in each group to represent one point of view only. Every type of institution is included, and *in addition* a special prize of three guineas will be awarded to the best account by the representative of any group, with illustrations. Any writer therefore may have a chance of winning five guineas, and the essays will reveal how few or how many institutional workers there are in the country alive to the spirit of personal service in themselves, and to a conscious understanding of it as the driving force in institutional life. **Prize essays must reach us not later than December 11, 1913.** They should be written plainly (preferably typed) on one side of the paper only, contain not more than 1,000 words, and be accompanied by the coupon from our current issue. The Editor's decision and awards are to be final and unquestioned. The prize essays will be published in the Christmas Number.

### MONDAY'S MEETING AT WORCESTER INFIRMARY.

A special meeting of the governors of Worcester General Infirmary was held on Monday last to consider several of the recommendations of the executive committee which were noted in The Hospital last week. The Mayor of Worcester, Mr. H. A. Leicester, was in the chair, and said he was delighted to see such a large meeting. The recommendation that the subscription for a letter of recommendation be 5s. 3d., and that eight such letters be required to admit an in-patient for one month, and one for each week of in-treatment after the first month, was carried. So was the proposal that the exclusive use of a bed be given to individuals, singly or in groups, who subscribe £50 a year. The qualifying subscription for a governorship was raised to two guineas. As regards the paying patients it was decided that beds be provided for those paying 31s. 6d. at the beginning of each week for maintenance and nursing, the patients to make their own arrangements with the honorary staff as to medical or surgical fees. Mr. Gostling's modified amendment that each paying patient should procure one letter, which was seconded by Dr. Crowe, was added to this resolution. A canvassing sub-committee was also formed to encourage the Saturday, Sunday, and Penny Monthly Funds. It was also decided to call a meeting of governors at the earliest date so to alter the rules as to allow the above proposals to come into effect with the New Year. Our view of these proposals was expressed in detail last week, but it appears to be hardly good management to have to call another meeting of governors to "alter the rules" which Monday's meeting of governors was summoned to sanction.

### THE SURPLUS INSURANCE FUNDS.

The vexed question of what is to be done with the medical benefit moneys of those insured who have not chosen a panel doctor has been practically settled by the Chancellor of the Exchequer, who in a letter to the London Insurance Committee has explained that he explicitly pledged this money to the men on the panels months ago. After this categorical assumption of responsibility, the Insurance Committees all over the country are almost certain to take Mr. George's view of the matter as an obligation of honour, and allot the accumulated sums, which in the aggregate are very large, to the panel doctors. It is true that the Chancellor's mere dictum does not override the law, and that no legal tribunal has yet decided the rights of the matter. But clearly any Committee which acts as the Chancellor suggests will have a very strong case against any possible objectors. The next difficulty which will confront the Committees is the method of distribution. We have on a previous occasion pointed out that injustice and unfairness will be exceedingly hard to avoid in dividing the spoil; and we note that Mr. George has no suggestion to offer on this point. The entire incident is instructive as one more proof of the straits to which the Chancellor was reduced a year ago in his tussle with the doctors and the artifices he resorted to in order to get the panels filled.

### A SIXTY-HOURS' WEEK FOR ATTENDANTS.

We note that the Lancashire Asylums Board, of which Sir Norval W. Helme, M.P., has been re-elected chairman, has rescinded the resolution passed a year ago that no increases in the maximum salaries of officers should be considered for three years, and a number of important increases have been granted. The salary of the senior assistant medical officer in future will start at £350 (an increase of £100) and rise by two annual increments to £450; the second assistant medical officer will start at £300 and rise to £350, while the pathologist and other assistant medical officers will begin at £250 and rise to £300. Unluckily the attendants were not so fortunate. Mr. C. G. B. Ellison's motion to refer back a recommendation that no reduction of attendants' hours of duty be entertained was defeated, and his argument that their request for a sixty-hour week was quite reasonable fell upon deaf ears. We are the more sorry because the committee's action in rescinding a barrier resolution on similar lines shows that the clock cannot be put back artificially nor the tide of progress be stemmed by order in advance, and, though no period of time was mentioned in the recommendation, against which Mr. Ellison struggled, for any body of skilled officers to have it officially stated that none of their demands on a certain head will be entertained must be exasperating and increase the discontent it is designed to allay. Such a consideration may at times be necessary for authorities to adopt in their inner consciousness, but to flaunt it in the faces of their subordinates by formal resolution or statement is unstatesmanlike and should be avoided.

### A ROYAL VISIT TO TUNBRIDGE WELLS.

On Thursday last week the Borough of Tunbridge Wells was honoured by a visit from H.R.H. Princess Alexander of Teck, who came to open a bazaar at the Great Hall in aid of the local Home in connection with Dr. Barnardo's Homes. Afterwards the Home was visited by the Princess on her way to luncheon at Eridge Castle. The Princess called at the General Hospital, Tunbridge Wells, and inspected the ward which was erected by the Borough to commemorate the Diamond Jubilee of Queen Victoria. This ward is exclusively for children, and is attractive, with large panels of tiles on the walls illustrating various nursery rhymes. The Princess spoke to each child in the cots, and gave, with much kindness, a flower to each from her bouquet. The cot endowed by the Burgesses of the town in memory of King Edward VII. came in for special notice. The reception was, at the wish of the Princess, of a quiet character, the hospital being represented by the Chairman, Mr. F. Wadham Elers, J.P.; the Matron, Miss A. M. Smith; House Physician and House Surgeon; and the Secretary, Mr. J. J. Webb. We may note in this connection that the committee of the Tunbridge Wells General Hospital have recently enlarged the scope of the dental department by electing six hon. dental surgeons, one of which attends each week-day for the purpose of extractions and conservative treatment. It is hoped that by this something will be done to improve the teeth of those who cannot afford to pay a qualified dentist's fee.

### THE SILENCE OF CAMBERWELL.

Several months have elapsed since attention was drawn in the columns of The Hospital to a serious outbreak of enteritis amongst the children in the Southwark Infirmary, which Dr. Bruce, the medical superintendent, attributed to the refuse of the Camberwell Borough Council's siding. Dr. Fletcher, an inspector of the Local Government Board, who did not entirely agree with Dr. Bruce, was yet of opinion that the siding was a source of danger to the public health. The Board supported his views, and asked the Council to take measures to discontinue the use of the siding. The Council, however, instructed its officials to report to the Public Health Committee, but that report has not yet been presented, nor has the Council taken any steps to meet the wishes of the Local Government Board. This prolonged delay has resulted in the Camberwell Borough Council receiving a letter of dissatisfaction from the Local Government Board. The letter states, on the report of their inspector, that the use of this siding was so undesirable that it should be discontinued. "The Board express their disapproval at the attitude of the Borough Council in the matter, and to press that immediate steps may be taken to abandon the use of this particular siding." This letter was relegated to the Public Health Committee without being communicated to the Council. Nothing has been done, and it would appear as if the Southwark Board of Guardians or the Local Government Board will have to take drastic action in order to get the abatement of what is a source of danger to the public health. The Council met on Wednesday, but the agenda paper, unless altered at the last moment, contained no reference to the refuse nuisance.

### MEASUREMENT OF FATIGUE IN SCHOOLS.

The very interesting question of fatigue formed part of Professor Stirling's Lady Priestley Memorial Lecture at a meeting of the National Health Society last week. After quoting Dr. Leonard Hill's observations on the value of oxygen in fatigue, the lecturer produced a table showing the relative fatigue produced by different subjects. In order of difficulty mathematics (standard), 100; Latin, 91; Greek, 90; gymnastics, 90; history and geography, 85; French and German, 82; natural history, 80; drawing and religion, 77. It does not need to be a Samuel Butler or an Anatole France to note the exquisite humour latent in these figures, which are valuable, we take it, rather as indicating the fatigue produced by the school methods of inculcating the subjects alluded to than the fatiguing properties of the studies themselves. For note that those placed highest in order of difficulty are those in which solid, definite patterns of knowledge have to be acquired, as in "sums" and equations; the grammar of dead languages; and muscular exercise. After these, with the curious exception of French and German, come history, natural history, drawing, and religion, the very subjects, in short, universally recognised by schoolboys as "slack" and semi-serious only. Even French and German, being taught by foreigners, naturally at a disadvantage in maintaining discipline, are explicable on this ground. "Religion" or "divinity" and drawing are recognised as the "slackest" hours of all, from the fact that either means the cursory questioning on parables and Bible stories, in which any schoolboy can pass the ridiculously loose standard of acquaintanceship demanded. Drawing, too, is always a time for fooling at school. Interest in these latter subjects usually cannot be awakened till what may be called the university age, and not always then. Interest in the former may be born of the necessity of mastering the technics of grammar, numbers, and the strain of the horse and parallel bars.

### DR. WALDO ON THE PANEL SYSTEM.

At an inquest at Southwark Coroner's Court last week Dr. Waldo, the coroner, made some criticisms on the panel system, which were provoked by a witness's description of the difficulty which he had found in obtaining medical assistance.

Dr. Waldo is reported to have said: "Now the panel system is in force I suppose it is very difficult in an emergency to find a doctor, and if you do find one his surgery is generally filled up with patients. Although the panel system," he added, "has its good points, there are some bad points, so they tell me. A doctor told me here the other day that he had 3,000 patients on his panel in addition to his private patients." The panel system can never do even bare justice to the insured until the numbers on each doctor's list are limited, and the highest figure at which this limit should be placed is 2,000. That limit political exigencies have allowed very frequently to be grossly overstepped, and the result is the frequency with which the panel system is brought into contempt at Coroners' Courts through investigations into deaths which might be prevented, or, at least, robbed of their bitterness.

### TWO PENAL CASES.

We alluded some time ago to a case in which a practitioner was fined for the issue of false statements upon forms of applications for passports. That case has now come before the General Medical Council, which, it is only fair to add, has not directed the erasure of the practitioner's name from the Medical Register. In another case brought before the Council, in which the complainants were the Medical Defence Union, a practitioner was struck off the Register for associating in practice with an unqualified and unregistered person. The Medical Defence Union has thus another successful action to its credit, to which its share lends an interest that does not otherwise invariably attach to the decisions of the Council, in so far as the hospital world as a whole is concerned. The question of the importance that practitioners must feel in respect of their statements in applications for passports has been sufficiently indicated in the proceedings of the Central Criminal Court. The temptation to regard them lightly, which is very common, has received a check, and that the responsibility involved may have every publicity which we can afford we allude to it once again this week.

### DEATH OF AN OBSTETRICIAN.

The death of Dr. Clement Dodson at the age of sixty-eight removes not merely a well-known obstetrician, but a medical man of high repute who threw himself vigorously into many interests where medical co-operation is required. Thus he was a vice-president of the League of Mercy, hon. surgeon-colonel of the Royal Army Medical Corps, Territorial Branch, and was in command of the 2nd London (City of London) General Hospital (Territorial Force). Himself the son of a surgeon, King's College School and St. Bartholomew's Hospital were succeeded by Aberdeen University, and in 1874 he graduated M.D. Returning to "Bart.'s," he took up the then new post of assistant physician-accoucheur, and his period of service in this position lasted for sixteen years. In Quain's Dictionary are several articles by him on his special subject, in which, by the way, he was examiner to the Universities of Aberdeen and Durham, while he was also president of the British Gynæcological Society.

### LATEST ADDITION TO PUBLIC HEALTH DIPLOMAS.

At the session of the General Medical Council last week the Public Health Committee presented a report recommending the Council to authorise the registration of the M.H. (Master of Hygiene) degree of the University of Liverpool as a qualification in Public Health. This was adopted, and is interesting as showing the demand that there is on all hands for Public Health officers, to which posts every new and duly authorised gateway tends to increase the candidatures. It is, in fact, a branch of medicine whose prestige even to-day is only an earnest of future importance.

### THE EXTENSION OF LEEDS INFIRMARY.

It is interesting to note that December has ushered in the beginning of the large extension scheme of the General Infirmary, Leeds. Last week the property on the site of the future infirmary buildings was offered for sale, and will be demolished, it is expected, by Christmas. As soon as this is complete the parallel scheme of the new thoroughfare will be begun. Our readers will remember what a great improvement to Leeds the combined schemes will be, and how the new road, cutting through congeries of present houses, will run straight from Woodhouse Lane to the Great Northern Railway Station. The infirmary buildings are not expected to be completed till the summer of 1916, but the practical start of this vast scheme is noteworthy, and will be an example to all of the effect on a community of public-spirited men and women of the Reports on Hospitals which have appeared in our columns during the last few years.

### THE BRITISH HOSPITAL FOR MENTAL DISORDERS AGAIN.

We understand that Dr. Bernard Hollander has resigned the post of physician to this institution on the ground that his suggestions for raising the institution from a dispensary to an "acknowledged" hospital met only with derision at the last committee meeting. On this occasion Dr. Hollander, moreover, expressed the view that the number of patients was so small as to make the expenses of management out of proportion to the benefit that the institution confers. The Chairman and another member of the executive committee also resigned at this meeting.

### MUNICIPAL HOUSES FOR DOCTORS.

The London County Council proposes to build on the Tottenham Housing Estate a house suitable for occupation by a medical practitioner, and thinks that the provision will benefit the tenants on the estate. This is an innovation, and the Council must secure the permission of the Local Government Board to the proposal. Assuming this to be gained, this will be the first time the Council has embarked upon a housing scheme for doctors. In this case, of course, the London County Council must do something of the sort, for it owns 200 acres at Tottenham, and the tenants naturally like to have a medical practitioner somewhere in the vicinity.

### ATTENDANTS' REDUCED HOURS AT CROYDON.

The hours and salaries, with a view to the reduction of the one and the raising of the other, of the attendants at Croydon Mental Hospital were discussed by the visiting committee of the institution last week. Councillor W. G. Stapleton supported the proposal of the house committee to reduce the hours, and said that even with the reduction they would be sixty-six a week, which was far too long for attendants at mental hospitals. Councillor H. T. Muggeridge referred to the night staff being on duty for ten and three-quarter hours. There were cases in which attendants had become patients, and he would rather be a patient than an attendant, for the patient was better looked after. Councillor W. B. Southwell said that they had endeavoured to meet the complaints in a reasonable spirit, and the recommendations of the house committee were carried. These included a present increase of wages amounting to £126 per annum, to cover the present deductions for superannuation, the allowance of one day a week to each attendant, and to each member of the male staff one evening a week after 5 P.M. These recommendations have been confirmed by the council, and once again we are glad to record the latest recognition of the severe strain of mental work upon the institutional staffs, whose conditions of service, even to-day, need, taking them as a whole, far greater improvement.

### FAMOUS PICTURES FOR A CORK HOSPITAL.

Miss Isabella Honan, of Cork, who left personal estate in the United Kingdom valued at £153,331, besides legacies of £15,000 to various Roman Catholic charities, has bequeathed her paintings of "The Cenci," the oil painting ascribed to Murillo, and the oil painting of "The Holy Family," by Raphael, to Honan's Home and Hospital, Cork. It may be wondered whether this bequest was intended to be in lieu of a cash legacy, since hospitals, for the most part, are ambitious to realise valuables. We had a recent example of this when a Midland hospital discovered an Old Master in its board-room, and immediately placed it to the treasurer's credit account.

### MEDICAL CANVASSING FOR INSURED PATIENTS.

At the conclusion of the session of the General Medical Council last week Mr. Verrall moved: "That this Council urges the Joint Commissioners under the National Insurance Act to exercise their power and influence to prevent the delivery in batches of forms of requests for transfer of an insured person to another practitioner, a course which directly leads to the encouragement of canvassing, and to insist on the obvious intention of the Act that such application should be made by the insured person on a form obtained by himself from his local Insurance Committee." This was carried unanimously. Mr. Verrall was elected a member of the National Insurance Act Committee. A letter addressed in March last to the Joint Insurance Commissioners was also read, in which the General Medical Council alluded to complaints of advertising for insured persons and their dependents which had been received. Some of these advertisements, it appears, were disguised under the apparent ægis of the Commissioners and local Insurance authorities, or in the supposed interests of approved societies and institutions. Warning notices have been issued disapproving of practitioners associating with medical aid associations which canvass or advertise, and as such associations have often sprung into existence in an attempt to hark back to the worst forms of contract practice, additional inducement to avoid them should be the result.

### CONFUSION IN COUNTY MEDICAL WORK.

Much discussion has arisen throughout the country as to the relation of the various county medical appointments to each other, and the question of dual officers has arisen. It would appear, at first sight, that there exists a distinction without much difference between a county medical officer who has also additional titles, such as that of chief tuberculosis officer, and one who has not. Upon the county medical officer, as the Council's adviser, fall the duties of organising and supervising (amongst other things) both the county scheme for dealing with tuberculosis and such of the school medical work as comes within the scope of the county authority. Beyond this the county medical officer should not be expected, or called upon, to go. The actual clinical and statistical tuberculosis work and the details of dispensary management should be left to the responsible tuberculosis medical officer, just as the routine examination of school children is deputed to the schools medical officer, each of them being specially appointed for the work involved. Where, however, the county medical officer is appointed a "chief" officer in such a special department it is a not unnatural assumption that he will be personally concerned in some of its special work, and that it is only necessary to appoint an "assistant" to him. Thus a less highly qualified, and less highly paid, post is created, to the disadvantage of the work. It is rarely the case that a county medical officer is able to devote time to the medical examination of patients or school children; so rapidly have his interests and responsibilities extended that his time is fully occupied with supervision, organisation, and advisory work, in which he has already, in many cases, medical assistance. The present is not the time to belittle either his work or that of his special departments: county medical work is undergoing development, already remarkable and likely in the near future to be extraordinary.

### NEW SUPERINTENDENT OF BRENTFORD INFIRMARY.

At a recent meeting of the Brentford Board of Guardians an intimation was received from Sir John Rose Bradford to the effect that he had examined Dr. J. B. Cook, the newly appointed medical superintendent, and was of opinion that he was physically fit for service and in sound health.

### AN UNGRATEFUL CHRISTMAS DUTY.

The time is approaching when hospital managers will be called upon to authorise and inspect decorations for the wards. This duty is always a difficult and ungrateful one, as the artistic ideas of the sisters often conflict with the opinion of the authorities as to what is safe from risk of fire and what decorations are least likely to cause undue dirt and damage to the walls and fittings. The favourite method of altering the appearance of the wards at Christmas time is to attach coloured shades of paper or other material to the electric-light fittings. This has the effect of giving a festive appearance without too much expenditure of labour and material, but the great disadvantage is that there is a distinct element of danger. A number of very serious fires in commercial establishments are caused by the explosion of an electric-light bulb and the ignition of adjacent inflammable material. The fact that there is any avoidable danger is sufficient to condemn this form of decoration in a building where there are a number of helpless people. Cotton-wool in any form must also meet with disapproval.

### PHTHISICAL PROBLEM IN LONDON INFIRMARIES.

For some months the Lambeth Board of Guardians have been considering the accommodation and equipment of their infirmary. It was felt by a section of the members that the only solution of the overcrowding was the erection of a new infirmary. The Board, however, have now come to the conclusion that additions to the existing buildings will meet the case. In the meantime the inmates of the infirmary and the workhouses are to be re-classified. It is indeed essential that something should be done at once. At the last meeting of the Guardians, Mr. F. Kinnaird said that phthisical cases, numbering 96, were mixed with general cases in the wards; and, although two years had elapsed since the disease was made notifiable, yet nothing had been done to isolate the patients. Dr. Baly, the medical superintendent, was of opinion they were spreading disease through the infirmary. He suggested that a disused building should be converted into a phthisical ward. The Rev. A. Amos said that the poor in Lambeth Infirmary were worse housed than those in Brixton Prison. The infirmary committee were instructed to take the matter in hand, and a resolution was adopted urging the Government to provide accommodation for all patients suffering from pulmonary tuberculosis in Poor-Law infirmaries. After such a statement as that of Dr. Baly, it would be inexcusable to await any such scheme.

### THE PUBLIC AND CROYDON HOSPITAL.

The financial position of the Croydon General Hospital as revealed by the report presented to the governors at their recent annual meeting is giving rise to some anxiety. The maintenance of the King Edward VII. Memorial Wing, opened last year, which has been fully occupied, considerably increased the expenditure, which reached a total of £6,238, while the ordinary income amounted to £5,220. This deficiency has been met by appropriating two legacies, life subscription, and a balance transferred from King Edward VII.'s Memorial Fund. In face of this large deficiency the committee are to be congratulated upon their determination not to close any of the beds, but to make every effort to raise the income to the required amount. The Chairman, Sir Frederick Edridge, in adressing the meeting, stated that subscriptions were being maintained. He even added that the public were taking an increased interest in the institution. Arrangements were in progress for extending the Hospital Saturday Movement; the result had so far been satisfactory, the collection during the past year amounting to over £500, and he did not feel that he would be too sanguine in anticipating that this return would ultimately be doubled. It was hoped by this and other means to maintain the hospital in a sound financial position.

### HOSPITALS AND TUBERCULOSIS DISPENSARIES.

The advantages of having a tuberculosis dispensary on the premises of or in close working connection with a hospital are very apparent. Apart from the benefit of additional expert advice which is always available in difficult cases, there is the obvious advantage of being able to refer patients to special departments without much risk of leakage occurring. To take one important example—the *x*-ray department. This aid to diagnosis is frequently employed. How much easier and more satisfactory it is to all concerned to be able to refer a patient straight away to the hospital radiologist! The medical officer can, if necessary, personally examine the patient with the screen and consult with the radiologist himself. The importance of dental treatment for tuberculosis patients is not sufficiently appreciated; but, here, for instance, the Chest Hospital in the City Road could offer great advantages, for this department does a very large amount of work on the dispensary patients, who can, moreover, be "followed up" most conveniently and assiduously. As an educative centre for nurses the Royal Hospital for Diseases of the Chest has gathered a large number of pupils, to whom a course of lectures is being given and facilities for practical work in the dispensary afforded. Certificates are awarded after an examination by members of the staff.

### MENTAL PATIENTS AND THE PANEL SYSTEM.

A very curious case, involving a panel practitioner's responsibility for his patient, and also the operation of the Lunacy Acts in respect of mentally diseased patients on the panel, was brought to light at the Hammersmith Coroner's Court last week. A young woman, aged twenty-seven, having committed suicide, an inquest was held at which her doctor, Mr. Butler, of Goldhawk Road, stated that she had shown symptoms of melancholia for some time, in consequence of which he had advised her relatives to send her to a mental hospital. This they did not consider her ill enough to do, and he understood that beyond advice he could do nothing, since the girl was a private patient, and, as such, beyond the reach of the relieving officer, who decides matters in the case of those who are destitute. The Coroner having suggested that Dr. Butler might have thrown up the case, as his advice was not attended to, the witness replied that the girl came under the Insurance Act, and he did not think he could give up attending her. The Insurance Act apparently had not provided for mental cases. Police evidence was also given to the effect that persons wandering and not under control had to be taken to the workhouse for medical examination as to their being sent to a mental hospital, but that a person in a private house was held to be under proper control. The questions thus arise: Can a panel practitioner refuse to attend a patient who, or whose relatives, refuse to take his advice? and, secondly, what does the Insurance Act accomplish for panel patients mentally diseased? In the case of such patients the possibility of their homes providing proper control may not be obvious, and in such cases, while avoiding the undesirable intervention of the Poor Law, what institutional treatment is to be provided for them? For, though the relieving officer may technically be free to take charge of any person, pauper or not, who is not under proper control, in practice it is only the destitute who come under his immediate influence.

### RESIGNATIONS AT CAMBERWELL INFIRMARY.

At the last meeting of the Camberwell Guardians the resignations of Dr. G. C. Soutter, assistant medical officer, and Staff Nurses E. E. Pollard, F. B. Cully, F. I. Richards, M. George, and M. B. Everett were accepted. The question of giving testimonials to Dr. Soutter and Dr. Mearns was referred to a committee.

### DREADNOUGHT HOSPITAL SUPERINTENDENTSHIP.

Mr. A. Gibson, F.R.C.S.Eng., has been appointed Professor of Anatomy at Winnipeg University, and is therefore resigning his post as medical superintendent at the Dreadnought Hospital, Greenwich. Mr. Robert S. Lawson, M.B., Ch.B., Edin., Demonstrator of Anatomy at Edinburgh University, has been appointed his successor.

### INSURANCE CERTIFICATES AND RUBBER STAMPS.

An interesting test case under the Insurance Act Regulations was decided on November 27 against the Prudential Approved Society for Women, which had refused to accept a panel doctor's certificate of incapacity for work because it was signed with a rubber stamp, and not in autograph. The first legal point raised was concerned with the jurisdiction of the Court to try the matter. The Prudential Society objected on the ground that by the Act the dispute should be submitted to arbitration, which had not been done. The Judge overruled this objection; but leave to appeal was given, and doubtless more will be heard of this point. A verdict for the plaintiff was then given, chiefly on the ground that the doctor had vouched to the Society for the genuineness of the case, and that thus the "other sufficient evidence" mentioned in the Act had been submitted. Unfortunately this does not dispose in the least of the question whether or not a rubber-stamp signature at the foot of a medical certificate is or is not in itself a valid claim to sickness benefit. Considering how exceedingly easy fraud would be if such signatures were accepted, common sense would seem to require that they should be refused; but then common sense and law are two totally distinct propositions.

### DEATHS, RETIREMENTS AND APPOINTMENTS.

The London Hospital has lost by death its oldest life governor, Sir Frederick Young, who joined in 1840, and also Major Sir William Evans Gordon, both of whom were some time members of its house committee. The Earl of Derby, owing to work which keeps him so much in the North of England, has resigned the office of treasurer to the London Hospital, and has been elected vice-president, together with the Duke of Devonshire and the Marquis of Salisbury. Dr. Bligh Wall, owing to the ill-health of his wife, has had to leave London, and has been succeeded as an assistant anæsthetist to the London Hospital by Dr. James D. Lyle, an old London student. Mr. Sowden Hills and Mr. Hugh Porteous have been appointed dental officers to attend four afternoons a week at the new dental department instituted at the London Hospital to take charge of the 1,540 children referred to it by the London County Council school doctors. The agreement already existing with the London County Council for the treatment yearly of 3,500 school children in the oral, 3,000 children in the ophthalmic, and 350 children in the skin department has been renewed for one year.

### THIS WEEK'S DRUG MARKET.

A slight improvement is noticeable in the Drug market, but business still continues quieter than is usual at this season. The chief movement that has taken place since the last report was written is a very considerable drop in the price of menthol. Opium has advanced in price partly as a result of speculative buying in the primary markets; higher prices are quoted for morphine, and the value of codeine is well maintained. Carbolic acid has a weaker tendency. An advance in the price of glycerine is not considered improbable. Higher prices for oil of sandalwood may be looked for. The price of balsam copaiba is well maintained, and the same applies to jalap. Podophyllum root is firm at the advanced rate. The decline in the price of castor oil has been checked and the value now has an upward tendency. The demand for cod-liver oil continues dull. Saffron has advanced in price. The value of cream of tartar has a slightly lower tendency. At the recent public sale of drugs in Mincing Lane menthol sold at a considerably reduced price; Japanese oil of peppermint was also much lower. Senna sold at dearer rates. Cardamoms were rather lower. East Indian ipecacuanha sold at cheaper rates.

